# The Role of P4HB and SOX4 in Prostatic Carcinoma and Their Clinical Significance

**DOI:** 10.30699/IJP.2024.2017851.3227

**Published:** 2024-02-15

**Authors:** Marwa Mohammed Dawoud, Noha Elkady, Rasha Adel Abdelmoneum, Ahmed S Ghonaimy, Dina Mohamed Allam

**Affiliations:** 1 *Department of Pathology, Faculty of Medicine, Menoufia University, Shibin Elkom, Egypt*; 2 *Department of Clinical Oncology and Nuclear Medicine, Faculty of Medicine, Menoufia University, Shibin Elkom, Egypt*; 3 *Department of Urology, Faculty of Medicine, Menoufia University, Shibin Elkom, Egypt*

**Keywords:** Diagnosis, ERG, Prostatic adenocarcinoma, Prognosis, P4HB, SOX4

## Abstract

**Background & Objective:**

Prostatic adenocarcinoma (PAC) is the second most prevalent cancer and the fifth leading cause of cancer death in men worldwide. Additionally, pathologists may face problems diagnosing it reliably and may need more than one marker. Thus, the search for new immunohistochemical biomarkers becomes mandatory. This study aims to investigate P4HB and SOX4 expression in prostatic carcinoma, their possible roles, and clinical significance.

**Methods:**

This retrospective study included fifty-six cases of PAC and an equal number of nodular prostatic hyperplasia (NPH) that were immunohistochemically stained by P4HB and SOX4. The results of expression were compared between PAC and NPH cases, followed by correlations with available clinicopathological parameters.

**Results:**

There was a highly significant difference between PAC and NPH regarding P4HB and SOX4 expressions in favor of PAC (both *P*<0.001). ROC curve analysis of the diagnostic power of P4HB showed 79% sensitivity, 76% specificity, and an area under the ROC curve of 0.845, while SOX4 showed (89%, 100%, and 0.946, respectively). P4HB and SOX4 expression showed a direct correlation (*P*<0.001). Moreover, the H-score of SOX4 expression showed a significant inverse relation with ERG expression (*P*=0.047). There was a significant correlation between P4HB and SOX4 and Gleason score (*P*<0.001). Moreover, P4HB expression was significantly associated with lymphovascular invasion (*P*=0.013), while SOX4 expression showed a significant association with perineural invasion (*P*=0.05).

**Conclusion:**

SOX4 and P4HB seem to have diagnostic and prognostic value in PAC. While there was a direct correlation between SOX4 and P4HB, an inverse relationship between SOX4 and ERG was detected.

## Introduction

Prostatic Adenocarcinoma (PAC) is the second most prevalent cancer and the fifth leading cause of cancer death in men worldwide (1). Approximately 7% of PAC patients are suffering from metastatic disease (2). In Egypt, the incidence of malignancy in male genital organs constitutes 1.34% of all malignancies, whereas prostatic carcinoma constitutes 61.63% (3). Although PAC is one of the cancers diagnosed at an early stage, the morbidity from its therapy is significant (4). 

Prostate-specific antigen (PSA) has been used as a tissue-specific biomarker for the diagnosis, screening, and management of PAC for decades (5). However, the diagnosis and prognosis of PAC are still a matter of controversy due to the presence of some mimic conditions at the level of serum and immunohistochemical biomarkers. For instance, PSA levels have been found to rise in some benign conditions such as prostatitis and NPH or in association with other cancers (6–8). Additionally, some procedures such as cystoscopy and colonoscopy may result in a rise in PSA (7,9). At the level of other immunohistochemical biomarkers, no single marker can used reliably for PAC diagnosis according to the diagnostic guidelines (10). Therefore, the search for new diagnostic and prognostic biomarkers becomes mandatory. 

Prolyl 4-hydroxylase beta polypeptide (P4HB) is a multifunctional protein belonging to the protein disulfide isomerase (PDI) family. In normal tissues, it catalyzes disulfide bonds in protein molecules. It acts as a molecular chaperone that helps to prevent endoplasmic reticulum (ER) stress due to the accumulation of misfolded proteins (11). On the other hand, its upregulation has been spotted in a variety of cancers, such as colon cancer, gastric cancer, bladder cancer, and clear cell renal cell carcinoma (11–14). In most of them, overexpression of P4HB may promote cancer cell survival, tumor angiogenesis, aggressiveness, immunosuppression, resistance to therapy, and decreased drug sensitivity (12,15,16). However, one research study has recently reported it in prostatic carcinoma (16). Thus, in the current study, we intend to explore the prospective role of P4HB in PAC. 

SOX4 (sex-determining region Y-box 4) is one of the SOX family of genes, including highly conserved genes coding 20 transcription factors that play essential roles during development processes through regulation of stemness and differentiation pathways (17). Regarding cancer studies, Sox4 overexpression has been reported in several types of cancer, including PAC (Moreno, 2020). All SOX4 expressions exhibited an association with poor prognosis (19).

SOX4 expression was found to be stimulated by many pathways. These pathways were commonly activated in a variety of cancers, including TGFβ signalling (20), Wnt (21), and phosphoinositol 3 kinase (PI3K) signaling (22). Although there is growing evidence linking P4HB with Wnt- β-catenin/Snail pathway signaling (23,24) and the TGFβ signaling pathway (16), there has been no study investigating the relationship between P4HB and SOX4 in PAC until now. Therefore, given the lack of studies evaluating the immunohistochemical expression of P4HB and SOX4 in PAC and their relationship with clinicopathological parameters, we designed this research to investigate P4HB and SOX4 expression in prostatic carcinoma, their possible roles and clinical significance and to test their hypothesized relationship.

## Material and Methods

Fifty-six cases (56) diagnosed as prostatic adenocarcinoma (PAC) and an equal number of nodular prostatic hyperplasia (NPH) were included in this retrospective case-control study. The paraffin blocks of the selected cases were retrieved from the archive of the pathology department of the Menoufia Faculty of Medicine after obtaining ethical approval (9/2023PATH11) from the Ethical Committee and in accordance with the Declaration of Helsinki. Case selection was done based on the availability of tissue blocks for re-cutting. The specimens were chips, cores, or open prostatectomy. 

The archive of the pathology was screened for all cases of NPH and PAC that were diagnosed between 2019 and 2022 which were 150. Inclusion criteria include all cases with available clinical data (age, clinical symptoms, serum PSA) and available paraffin tissue blocks for re-cutting. Exclusion criteria include patients with double primaries, unavailable clinical data or tissue blocks, and scanty tissue cores not suitable for immunostaining. After applying inclusion and exclusion criteria, 38 cases were excluded due to the unavailability of tissue blocks for re-cutting or clinical data, the remaining 112 cases were eligible and were allocated as participants to be included in the study and analyzed.

**Histopathological Evaluation**

Sections were cut from each block stained with hematoxylin and eosin and examined using a light microscope to confirm the diagnosis and evaluate different histopathological features such as percentage of the tumor, Gleason grade, group grade, lymphovascular invasion, perineural invasion, and prostatic intraepithelial neoplasia (PIN). 

**Immunohistochemical Staining**

Four-micron thick sections were mounted on positively charged slides; then they were immunohistochemically stained using the streptavidin-biotin amplified system in which diaminobenzidine (DAB) was used as a substrate/chromogen. The slides were deparaffinized in xylene, followed by rehydration. The sections were boiled in citrate buffer saline for antigen retrieval. Primary antibodies were added to the sections and incubated overnight. Then, a detection kit (Envision, FLEX, code 8002, Dako) was added. P4HB antibody (rabbit polyclonal antibody, A0692, concentrated 1:150, Wuborn, USA) and SOX4 antibody (Rabbit polyclonal antibody, RB-9249-R7, ready to use, Thermo Fisher Scientific, Fremont, USA) were used. Placental tissue and breast carcinoma sections were selected as positive control together with a negative control slide.

*Immunostaining assessment:*

The finally included cases (n=112) were immunohistochemically stained, scored, and analyzed. The expression of P4HB and SOX4 were evaluated by two pathologists. Their expressions were assessed in terms of status (positive or negative), the intensity of staining, and the percentage of positive cells.

Then, the expression was semi-quantitatively scored using H-score and Immuno-Reactive Score (IRS). In the H score, the intensity of staining was scored as follows: 0 (no staining); 1 for low (pale brown), 2 for moderate (faint brown), and 3 for strong or high (dark brown). The percentage of positively stained cells was also evaluated as the number of positive cells divided by the whole number of cells. The H-score was calculated by multiplying the above two scores. 

In the immunoreactive score, the intensity of staining is scored 0-3, while the percentage of positive cells is scored 0-4. The score is then calculated by multiplying both scores (25).

Clinical data of the cases including age and prostatic specific antigen level (PSA) were collected from patients’ records.

**Statistical Analysis**

Data from all included cases (n=112) were collected, tabulated, and statistically analyzed using SPSS (statistical package for the social science software) version 26 on an IBM-compatible computer. Two types of statistics were done: 

Descriptive statistics: were expressed as number and percentage (No & %) for qualitative data and mean, standard deviation (SD), range, median, and interquartile range (IQR) for quantitative data.

Analytic statistics: Chi-squared test (χ2), Mann Whitney (U) test, Kruskal Wallis test (K), Student T test (t), One Way ANOVA (F), and Spearman correlation (r). 

Receiver operator characteristics (ROC) with respective points of maximal accuracy for sensitivity and specificity were generated to determine biomarker diagnostic performance. The area under the ROC curve (AUROC) measures the accuracy of the test. An area of 1 represents a perfect test; an area of 0.5 represents a worthless test. 0.90-1 = excellent (A); 0.80-0.90 = good (B); 0.70-0.80 = fair (C); 0.60-0.70 = poor (D), 0.50-0.60 = fail (F). Sensitivity is the proportion of patients with disease who test positive. Specificity is the proportion of patients without disease who test negative. The predictive value of a positive test is the proportion of patients with positive tests who have the disease. The predictive value of a negative test is the proportion of patients with negative tests who do not have the disease.

A P-value≤0.05 is considered statistically significant, and a P-value< 0.001 is considered highly significant.

## Results

This retrospective study was conducted on 112 prostatic biopsies, including 56 Prostatic Adenocarcinoma (PAC) cases and 56 cases of Nodular prostatic hyperplasia (NPH). The clinicopathological characteristics of the studied cases are shown in Table 1.

**Table 1:** Clinicopathological parameters in the studied cases (n=112).

Parameters	Prostatic adenocarcinoma )n=56)		Nodular prostatic hyperplasia (n=56)	
**No**	**%**	**No**	**%**
Age Mean ±SD Range (Min-Max) Median (IQR)	66.98 ±7.37 54-86 65 (61.25-73.75)		65.79 ±7.37 52-83 65 (61.25-72.75)	
Specimen Chips Cores Open	15 39 2	26.8 69.6 3.6	48 8 0	85.7 14.3 0.0
PSA Mean ±SD Range (Min-Max) Median (IQR)	87.68 ±42.94 38-240 77 (60.475-100)		10.36 ±7.89 2-30 7 (4-17)	
Cancer percentage Mean ±SD Range (Min-Max) Median (IQR)	79.12 ±30.93 1-100 90 (70-100)		---------------	
Gleason grade 6 7 8 9	6 30 9 11	10.7 53.6 16.1 19.6	---------------	
Group 1 2 3 4 5	6 15 15 9 11	5.4 13.4 13.4 8.0 9.8	-------------	
Lympho-vascular invasion Positive Negative	13 43	23.2 76.8	--------------	
Perineural invasion Positive Negative	16 40	28.6 71.4	-------------	
PIN Positive Negative	2 54	3.6 96.4	-------------	

SD: Standard deviation, IQR: Inter quartile range

**Results of Immunohistochemistry**

**Fig. 1:**
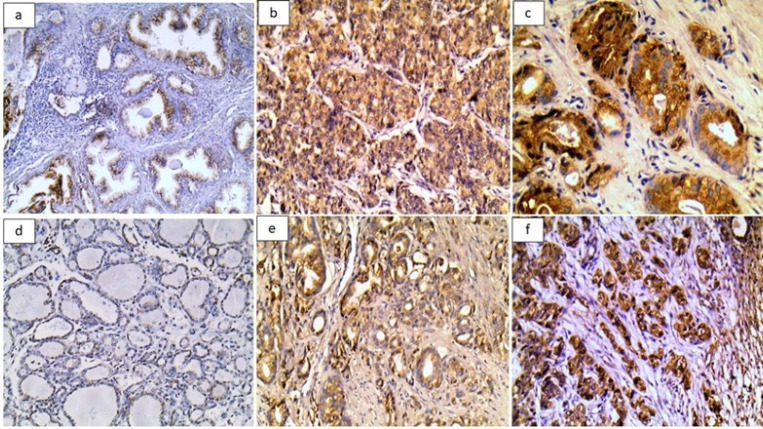
Immunohistochemical staining of P4HB, and SOX4 in the studied cases. a. Mild P4HB expression in glandular epithelium in nodular prostatic hyperplasia. b. Moderate P4HB expression in tumor cells in prostatic carcinoma. c. Strong P4HB expression in tumor cells in Grade 5 prostatic carcinoma. d. Negative expression of SOX4 in nodular prostatic hyperplasia. e. Mild to moderate SOX4 expression in tumor cells of PAC. f. Strong SOX4 expression in tumor cells in Grade 5 prostatic carcinoma.

Immunohistochemical expression of P4HB in cases of NPH was positive in 66.1% compared to 100% in PAC, resulting in a highly significant difference (*P*<0.001). Additionally, the H score and IRS revealed the same results (both *P*<0.001) (Table 2) (Figure 1 (a-c)).

The expression of SOX4 in cases of NPH was entirely negative. Meanwhile, it was detected in the nuclei of malignant cells in 89.3% of PAC cases, with a highly significant difference (*P*<0.001) (Table 2) (Figure 1 (d-f)). 

**Table 2:** Comparison between the studied groups regarding P4HB, SOX4, and ERG expression in the studied cases (n= 112).

Parameters	Prostatic adenocarcinoma )n=56)		Nodular prostatic hyperplasia (n=56)		Test of significance	P-value	Odds ratio (95% CI)
**No**	**%**	**No**	**%**
P4HB							
Status Positive Negative	56 0	100.0 0.0	37 19	66.1 33.9	χ2=22.882	<0.001*	0.60 (0.50-0.70)
Intensity Low Moderate High	7 17 32	12.5 30.4 57.1	(n=37) 22 12 3	59.5 32.4 8.1	χ2= 30.02	<0.001*	4.452 (1.443-13.738) 33.524 (7.806-143.973
H score Mean ±SD Range (Min-Max) Median (IQR)	200.18 ±67.76 60-280 210 (160-270)		107.57 ±55.65 20-240 100 (60-155)		U =5.648	<0.001*	1.021 (1.012-1.029)
IRS Mean ±SD Range (Min-Max) Median (IQR)	9.20 ±3.10 2-12 9.5 (8-12)		5.00 ±2.59 0-12 4 (3-8)		U =5.493	<0.001*	1.021 (1.012-1.029)
SOX4							
Status Positive Negative	50 6	89.3 10.7	0 56	0.0 100.0	χ2=90.323	<0.001*	-------
Intensity Low Moderate High	(n=50) 9 15 26	18.0 30.0 52.0	00 00 00	00 00 00	--------	----------	------
H score Mean ±SD Range (Min-Max) Median (IQR)	152.50 ±88.53 0-280 180 (67.5-210)		000 000 000		U= 8.952	<0.001*	------
IRS Mean ±SD Range (Min-Max) Median (IQR)	6.98 ±3.93 0-12 8 (3-9)		000 000 000		U= 8.963	<0.001*	--------

* Statistically significant, SD: Standard deviation, IQR: Inter quartile range, χ2: Chi-square test, U: Mann Whitney U test.

**The Diagnostic Power of P4HB and SOX4 in the Prediction of Prostatic Adenocarcinoma**

**Fig. 2:**
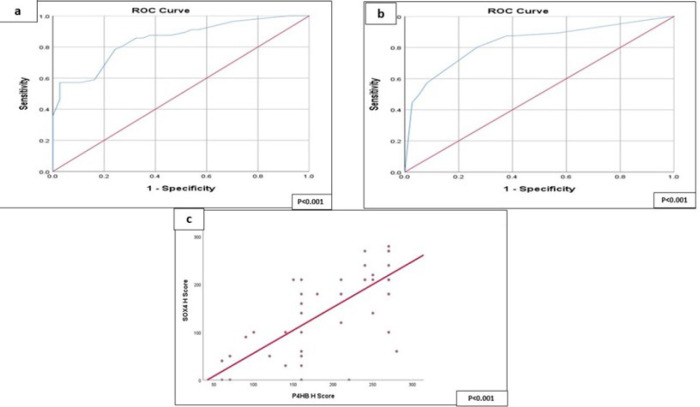
(a) ROC curve of P4HB H score (AUC= 0.845, 95% CI=0.768-0.922). (b) ROC curve of SOX4 H score (AUC= 0.946, 95% CI=0.898-0.995) (c) Scatter plot of P4HB -H Score in relation to SOX4 H score in the studied participants with prostatic adenocarcinoma (*P*<0.001).

For testing a possible diagnostic power of P4HB and SOX4 in the prediction of PAC, a ROC curve was implemented that denoted good diagnostic validity of P4HB and SOX4 in PAC (*P*<0.001) (Table 3) (Figure 2a and 2b). 

**Table 3:** Diagnostic accuracy of P4HB and SOX4 in prediction of prostatic adenocarcinoma.

P4HB H score		SOX4 H score	
AUC	0.845 (B)	AUC	0.946 (A)
SE	0.039	SE	0.025
P-value	<0.001**	P-value	<0.001**
95% CI	0.768-0.922	95% CI	0.898-0.995
Cut off point	155	Cut off point	15
Sensitivity	79%	Sensitivity	89%
Specificity	76%	Specificity	100%
PPV	83%	PPV	100%
NPV	80%	NPV	90%
Accuracy	81%	Accuracy	95%

Statistically significant, SE: Standard error, CI: Confidence interval, ** = highly significant

AUC (Area under the ROC curve): 0.90-1 = excellent (A), 0.80-0.90 = good (B), 0.70-0.80 = fair (C), 0.60-0.70 = poor (D), 0.50-0.60 = fail (F)

There were highly significant correlations between the H Score of P4HB and SOX4 and some clinicopathological parameters of aggressive tumor traits (Table 4).

There was a highly significant correlation between P4HB and SOX4 expression scored by H score (*P*<0.001) (Figure 2c)

Data about ERG expression that was tested previously on the same cohort of cases was available (26). The current study revealed a statistically significant inverse correlation between ERG expression and SOX4 scores (H score *P*=0.047, IRS *P*=0.043) (Table 5).

**Table 4:** Relation of H Score of P4HB and SOX4 to clinicopathological parameters in the studied participants with prostatic adenocarcinoma. (n=56)

Parameters	P4HB H score Mean ±SD	Test of sig.	P-value	SOX4 H score Mean ±SD	Test of sig	P-value
Age	*r *= - 0.067		0.622	*r* = - 0.158		0.246
Specimen Chips Cores Open	182.67 ±70.66 209.49 ±66.77 150.00 ±14.14	K= 3.967	0.138	146.00 ±84.49 154.87 ±92.36 155.00 ±77.78	K =0.308	0.857
PSA	*r* =0.120		0.378	*r *=0.241		0.073
Cancer percentage	*r* =0.085		0.543	*r* =0.022		0.871
Gleason grade 6 7 8 9	75.00 ±22.58 182.67 ±45.93 264.44 ±14.24 263.64 ±15.67	F=47.206	<0.001*	23.33 ±25.82 140.00 ±70.76 213.33 ±66.14 207.27 ±87.42	K =22.301	<0.001*
Group 1 2 3 4 5	75.00 ±22.58 152.00 ±20.07 213.33 ±44.19 264.44 ±14.24 263.64 ±15.67	F= 68.547	<0.001*	23.33 ±25.82 112.67 ±72.36 167.33 ±59.46 213.33 ±66.14 207.27 ±87.42	K =25.408	<0.001*
Lymphovascular invasion Positive Negative	226.67±69.71 187.63±63.94	U =2.471	0.013*	161.33±94.48 149.27±87.25	U =0.635	0.525
Perineural invasion Positive Negative	208.67±84.50 197.07±61.45	U =1.082	0.279	188.89±74.35 135.26±90.34	U =1.957	0.05*
PIN Positive Negative	190.00 ±42.43 200.56 ±68.75	t = 0.336	0.786	70.00 ±98.99 155.56 ±87.65	U =1.381	0.203

SD: Standard deviation, r: Spearman correlation, K: Kruskal Wallis test, F: One Way ANOVA test, U: Mann Whitney U test, t: Student t-test, * = significant, ** = highly significant

**Table 5:** Relation between the status of ERG and H score and IRS of both P4HB and SOX4 in the studied participants with prostatic adenocarcinoma. (n=56)

ERG expression	H score Mean ±SD	Test of sig.	P-value	IRS Mean ±SD	Test of sig	P-value
P4HB						
Positive Negative	184.14 ±77.39 217.41 ±51.63	U =1.359	0.174	8.38 ±3.58 10.07 ±2.24	U =1.662	0.097
SOX4						
Positive Negative	131.38 ±88.79 175.19 ±84.00	U =1.986	0.047*	5.97 ±3.93 8.07 ±3.70	2.019	0.043*

* Statistically significant, SD: Standard deviation, U: Mann Whitney U test

## Discussion

This study aimed to evaluate the expression of P4HB and SOX4 in prostatic carcinoma and investigate their possible diagnostic or prognostic roles. The current study revealed that P4HB and SOX4 were significantly expressed in PAC compared to NPH which suggests their oncogenic role in prostatic carcinoma and strongly indicates its diagnostic capability which was further confirmed by ROC curve analysis. Their expression in PAC was also associated with aggressive tumor features such as high grade, lymphovascular invasion, and perineural invasion which pointed to their possible roles as prognostic markers as well. 

The poor prognosis and resistance to therapy associated with most cases of PAC (27) necessitate studying different molecular abnormalities associated with PAC aiming at identifying novel diagnostic and prognostic molecules that can also serve as targets of therapy. 

Autophagy is a catabolic process that decays different cytoplasmic materials or malfunctioning organelles and proteins by lysosomes (28)). That is why autophagy is important for regulating normal cell homeostasis. However, the role of autophagy in cancer is still a matter of controversy. In a context-dependent manner, some studies illustrated its prospected tumour-suppressive role (29), while others proved a cancer-promoting role and drug resistance (30). One of the newly recognized autophagy mediators is P4HB. In addition, cancer stem cells (CSC) play an important role in cancer development and progression and SOX4 is one of the CSC regulators. The roles of P4HB and SOX4 have not been extensively investigated in PAC yet, this has encouraged us to test their role in carcinogenesis and evaluate their diagnostic power. 

 P4HB expression was significantly expressed in different cancers, such as colon cancer, gastric cancer, bladder cancer, and clear cell renal cell carcinoma (11–14). Although expression of P4HB is expected in normal tissues due to its role in homeostasis (28), overexpression of P4HB has been explained by increased protein demand and synthesis in cancer cells, which is one of the fundamentals of carcinogenesis (31,32). Since P4HB which is an ER chaperone whose primary function is to prevent ER stress caused by the accumulation of misfolded protein molecules, increased protein synthesis results in the activation of the unfolded protein response (UPR) (33). In PAC, the UPR has been shown to enhance protein folding, translation, and, hence, PAC cells' survival (34). Therefore, our novel results support the oncogenic role of P4HB in PAC was supported by the previous reports by Jin et al. who revealed that P4HB is implicated in prostate carcinogenesis (35) however, more studies are warranted to validate this result as this is the first study to investigate P4HB diagnostic power in PAC.

Regarding the prospective prognostic power of P4HB in PAC, significant direct correlations were revealed between P4HB expression and high Gleason score as well as the presence of vascular invasion. Both are well-established factors of poor prognosis in PAC (36). This direct correlation is not surprising due to the previous reports suggesting its relationship with epithelial-mesenchymal transition (EMT). For instance, MA et al. demonstrated that P4HB expression impacts invasion and migration as well as chemosensitivity of liver cancer cells in in-vitro via stimulation of EMT. The researchers showed that this might occur throughout triggering nuclear β‑catenin, which is a main component of the Wnt signaling pathway ((37)). EMT is a complex process wherein cells downregulate epithelial characteristics and acquire mesenchymal traits, including mobility and invasiveness ability, leading to tumor progression and metastasis (38) Additionally, multiple studies linked EMT to drug resistance in several cancers (39). Furthermore, proteomic and protein functional studies revealed a strong association between high levels of PDIs, a family protein disulfide isomerase to which P4HB belongs, and lymphatic invasion in cancers (40). A recent study suggested that P4HB may be used as a potential prognostic marker in PAC that can predict radiotherapy resistance. The authors showed that P4HB can potentially affect prostate cancer cells' proliferation (16). These shreds of evidence collectively confirm our findings about the poor prognostic role of P4HB in PAC. Therefore, it can be a surrogate biomarker for target therapy in PAC. 

SOX4 has been detected in several cancers (19) including PAC, where it was reported in very few studies (18). Its functions are regulation of stem cell pathways, cellular proliferation, migration, and invasion of tumor cells (41–43). Our results suggested the oncogenic role of SOX4 in prostatic carcinoma which is in concordance with the previous report by Bilir et al who observed that SOX4 is implicated in prostate carcinogenesis in cases initiated by homozygous loss of PTEN (35) 

In terms of the correlation between SOX4 expression and clinicopathological parameters of prognostic importance, there was a positive correlation between the high Gleason score and the presence of perineural invasion. This association confirms that SOX4 is one of the prognostic biomarkers denoting bad prognosis in PAC. Being a direct target gene of C-MYC, it is not surprising to detect a positive association of SOX4 expression to bad prognosis in PAC (44), where C-MYC was consistently reported to be an independent prognostic biomarker in PAC (45,46)

Furthermore, some researchers like Liu and his colleagues reported SOX4-induced EMT in PAC. They also demonstrated that SOX4 knockdown suppressed proliferation and migration and increased the invasive capability of prostate cancer cell lines in vitro (47,48). Consequently, de-novo expression of SOX4 in PAC cases not only helps confirm the diagnosis of PAC but also may define a subset of cases with poor prognosis and render SOX4 a surrogate therapeutic target for PAC. 

In previous studies, SOX4 expression was found to be stimulated by many pathways. These pathways were commonly activated in a variety of cancers, including TGFβ signaling (20), Wnt (21), and PI3K signaling (22). On the other hand, there is growing evidence linking P4HB with Wnt- β-catenin/Snail pathway signaling (23,24) and the TGFβ signaling pathway (16). Additionally, both P4HB and SOX4 have been accused of orchestrating EMT (37,47). Thus, we were interested to discover the probable relation between P4HB and SOX4. Interestingly, when we studied the correlation between P4HB and SOX4 in our sample of PAC cases, there was a positive correlation. These shreds of evidence suggest that dual inhibition of both may dramatically improve the prognosis of PAC patients. 

Luckily, the data about the ERG expression for the same cohort of cases were available (26). Thus, we were interested in looking at the proposed correlation between expressions of P4HB, SOX4 and ERG. The main reason for this is that ERG is one of the surrogate genes involved in the new molecular classification of PAC that has been anticipated to affect prognosis (49). Herein, we demonstrated its inverse correlation to SOX4, which supports the poor prognosis implication of SOX4 in PAC. Mainly since we previously reported the direct association with good tumor characteristics in PAC, such as low Gleason and low Ki76 scores in the same cohort of patients (26). Despite this, some research showed that both ERG and SOX4 promote EMT in PC cells; therefore, they may confer poor prognosis (50). However, the prognostic implication of ERG expression in PAC is still a matter of controversy. Whereas some studies showed its direct association with EMT and poor prognosis (50), others found that ERG positivity was not linked to either aggressive tumor characteristics or a worse prognosis (51). This controversy could be attributed to genetic variability, taking into consideration the similar results reported by an Egyptian group of researchers (52). 

Briefly, SOX4 and P4HB can be of benefit in the diagnosis of PAC so that they can be added to the diagnostic panel. Furthermore, they may be considered as biomarkers for aggressive PAC as the expression of both seems to confer poor prognostic traits in PAC cases due to their direct correlations to advanced Gleason score, perineural invasions, and lympho-vascular invasions as well as the inverse correlation of SOX4 with ERG. Moreover, the two markers seem to have a positive correlation however, the underlying mechanism was not specified. 

The absence of funding was the main limitation in this study, in addition to the limited techniques and microscopes that hindered us from conducting large-scale research using more advanced testing. Moreover, due limited number of cases with available paraffin blocks and clinical data, all cases fulfilling the inclusion criteria were included for analysis without matching both groups. Additionally, the lack of survival data impeded us from conducting survival analysis. 

Finally, diagnostic panels including P4HB and SOX4 can be constructed and tested on large-scale cases. Moreover, further investigations using molecular testing are recommended to validate the diagnostic and prognostic roles of these markers, explore the precise mechanism underlying the association between both markers and test the probability of using them as target therapy in PCA.

## Conclusion

This study provides novel insights for P4HB and SOX4 diagnostic and prognostic roles in PAC that could define a subset of patients with aggressive behaviour who may require specific treatment modality via targeting both genes/proteins, leading to breakthroughs in personalised medicine in PAC patients.
